# Changes in Otx2 and Parvalbumin Immunoreactivity in the Superior Colliculus in the Platelet-Derived Growth Factor Receptor-****β**** Knockout Mice

**DOI:** 10.1155/2013/848265

**Published:** 2013-11-11

**Authors:** Juanjuan Zhao, Susumu Urakawa, Jumpei Matsumoto, Ruixi Li, Yoko Ishii, Masakiyo Sasahara, Yuwen Peng, Taketoshi Ono, Hisao Nishijo

**Affiliations:** ^1^System Emotional Science, Graduate School of Medicine and Pharmaceutical Sciences, University of Toyama, Toyama 930-0194, Japan; ^2^Department of Anatomy, Histology and Embryology, Shanghai Medical School, Fudan University, Shanghai 200032, China; ^3^Department of Judo Physiotherapy, Graduate School of Medicine and Pharmaceutical Sciences, University of Toyama, Toyama 930-0194, Japan; ^4^Department of Pathology, Graduate School of Medicine and Pharmaceutical Sciences, University of Toyama, Toyama 930-0194, Japan

## Abstract

The superior colliculus (SC), a relay nucleus in the subcortical visual pathways, is implicated in socioemotional behaviors. Homeoprotein Otx2 and **β** subunit of receptors of platelet-derived growth factor (PDGFR-**β**) have been suggested to play an important role in development of the visual system and development and maturation of GABAergic neurons. Although PDGFR-**β**-knockout (KO) mice displayed socio-emotional deficits associated with parvalbumin (PV-)immunoreactive (IR) neurons, their anatomical bases in the SC were unknown. In the present study, Otx2 and PV-immunolabeling in the adult mouse SC were investigated in the PDGFR-**β** KO mice. Although there were no differences in distribution patterns of Otx2 and PV-IR cells between the wild type and PDGFR-**β** KO mice, the mean numbers of both of the Otx2- and PV-IR cells were significantly reduced in the PDGFR-**β** KO mice. Furthermore, average diameters of Otx2- and PV-IR cells were significantly reduced in the PDGFR-**β** KO mice. These findings suggest that PDGFR-**β** plays a critical role in the functional development of the SC through its effects on Otx2- and PV-IR cells, provided specific roles of Otx2 protein and PV-IR cells in the development of SC neurons and visual information processing, respectively.

## 1. Introduction

The mammalian superior colliculus (SC) is a seven-layered structure on the roof of the midbrain [1] and can be divided into two functionally distinct units: a superficial subdivision and a deep subdivision [2, 3]. The superficial layers zonale (ZO), superficial gray (SGR), and optic (OP) layers receive their major inputs from the retina and the visual cortex and are involved exclusively in visual information processing. In contrast, the four deep layers intermediate gray (IGR), intermediate white (IW), deep gray (DGR), and deep white (DW) layers, which receive auditory, somatic, and visual inputs from numerous cortical and subcortical areas, are involved in the control of orientating behaviors of head, eye and ear [1, 4–6]. Furthermore, recent studies suggest that the subcortical visual pathway including the retina, SC and pulvinar is implicated in socioemotional behaviors [7, 8].

Several studies have used parvalbumin (PV) as markers to reveal details of the parallel subcortical pathways involved in visual processing [9]. In the primate lateral geniculate nucleus (LGN), PV is found in the relay cells in the P and M pathways [9–11]. In the cat SC, Mize et al. [12] found a band of PV-immunoreactive (IR) cells (PVcells) in the deep SGR and upper OP, a region of the cat SC that receives inputs from Y retinal ganglion cells [13]. In addition, PV is also one of the biochemical markers of the matrix surrounding some of the IGR compartments in the SC [14]. Other studies have associated PV with specific firing patterns of neurons, which are often found in “fast spiking” neurons with nonadapting trains of action potentials. It has been suggested that PV might regulate a Ca^2+^-activated K^+^ channel involved in spike adaptation [15], and a particular type of K^+^ channel was developmentally regulated with PV and was expressed in about 80% of the PV-containing GABAergic interneurons in the rat hippocampus [16]. Furthermore, deficits in these PV-containing GABAergic interneurons were associated with schizophrenia and autism with social deficits [17, 18], and the number of PV-positive neurons was reduced in the SC of an animal model of autism with prenatal valproic acid exposure [19].

 The Otx2 homeobox gene is a vertebrate orthologue of the *Drosophila* orthodenticle gene [20–22]; members of this orthology group play a fundamental role in development of photoreceptors Otx2 and Crx (cone-rod homeobox) and rostral brain regions (Otx1 and Otx2) [23, 24]. Otx2 also appears to play a role in development and function of the retina, in which the gene is expressed at both prenatal and postnatal stages [25–29]. In addition, Otx2 coordinates postnatal PVcell maturation and activates visual cortical plasticity [30]. Otx2 is strikingly restricted to relay centers in the primary visual pathway at birth before PV-cells are evident [31], including the retina, LGN, and visual cortex (V1). Furthermore, exogenous Otx2 infusion accelerated PV-cell development in the V1 area [30]. These findings indicate that Otx2 is related to development of the visual system. However, to our knowledge, no information is available concerning Otx2 expression in the retinocollicular (retinotectal) visual pathway in adult mice. It is possible that Otx2 may control functional features of SC neurons in adult mice.

Platelet-derived growth factor (PDGF) was originally reported as a substance in platelets that promote growth of tissue culture cells [32]. However, the factor is recently reported to act as a neuroprotective factor in the central nervous system (CNS) [33–36]. PDGF is also involved in the regulation of cell growth and differentiation during embryonal development [37–39]. The family of platelet-derived growth factors (PDGFs) comprises 4 members—PDGF-A, -B, -C, and -D—that are assembled from disulfide-linked homo- or heterodimers of 2 distinct but related chains (PDGF-AA, -AB, -BB, -CC, and -DD). Two receptor subtypes of PDGF (PDGFR-*α* and -*β*) can form mature dimeric receptor complexes that can bind to ligands with different affinities [40]. PDGFR-*α* is largely expressed in oligodendroglial progenitors, while PDGFR-*β* is predominantly expressed in neurons [41] and upregulated in the neonatal rat brain [42]. PDGF-BB that specifically binds to PDGFR-*ββ* is abundantly expressed in neurons and is upregulated in neonatal brains [33, 43, 44]. PDGF-B exerts neurotrophic effects on *γ*-aminobutyric acid GABA ergic neurons [42, 45]. Altering PDGFR-*β* may result in abnormalities during the development of the CNS [46, 47]. Consistently, conditional knockout (KO) mice with suppressed expression of neuronal PDGFR-*β* in the Cre/loxP system (PDGFR-*β* KO mice) displayed autistic/schizophrenic traits, especially deficits in socioemotional behaviors [48].

Taken together, there is a possible link among PV, Otx2, and PDGFR-*β*, and behavioral deficits in the PDGFR-*β* KO mice might be ascribed to changes in PV and Otx2 in the SC. In the present study, to investigate the role of PDGFR-*β* in Otx2 and PV expression in the SC, we analyzed immunohistochemical alterations in Otx2 and PV expression in the SC of PDGFR-*β* KO mice.

## 2. Materials and Methods

### 2.1. Generation of the Conditional PDGFR-*β* Knockout Mice

The Cre/loxP system was used to develop conditional PDGFR-*β* KO mutants. A previously established mutant mouse line was used, in which exons 4–7 of PDGFR-*β*, which encode the extracellular domain of the PDGFR-*β* protein, were flanked by 2 loxP sequences (floxed) positioned in introns 3 and 7 [49]. After Cre-mediated recombination, deletion of the loxP-flanking region and resulting frame shift mutation in the adjoining 3′ region occurred in PDGFR-*β*. To obtain conditional PDGFR-*β* KO, we then crossed mutant mice harboring the PDGFR-*β* floxed allele and those expressing Cre recombinase under the control of the nestin promoter and enhancer (nestin-Cre+ mouse, The Jackson Laboratory, Bar Harbor, ME, USA) as previously described [50, 51]. Before this cross, both mutant mice harboring floxed PDGFR-*β* and nestin-Cre+ were outbred to the mice of C57BL/6J (B6/J) strain for 14 generations to replace the genetic background of our mutant mice with that of the B6/J strain. In the present study, the following 2 types of 22 to 29 weeks old male mice were used: mice with the Cre transgene and floxed PDGFR-*β* (PDGFR-*β* KO mice) and mice without the Cre transgene but with floxed PDGFR-*β* (control mice).

Genotypes were confirmed by PCR of tail DNA, using oligonucleotide primers pairs for floxed PDGFR-*β* and for the Cre transgene as described previously [50]. The genotyping was confirmed by Western blot of the total lysates of the adult mouse brains to show that the PDGFR-*β* expression decreased to undetectable levels in the PDGFR-*β* KO mice compared with that in the control mice [50].

In the present study 6 control mice (24–28 weeks old, 22–29 g body weight) and 4 PDGFR-*β* KO mice (24–28 weeks old, 22–29 g body weight) were used. All mice were housed in individual cages in a temperature-controlled environment with a 12/12 h light/dark cycle (lights were turned on and off at 08:00 and 20:00, resp.). Food and water were supplied ad libitum. Mice (10 to 16 weeks old) were handled for 3 consecutive days before the start of the experiments. All experimental protocols were performed in accordance with the guidelines for Care and Use of Laboratory Animals approved by the University of Toyama and the National Institutes of Health's Guide for the Care and Use of Laboratory Animals, and approved by the Committee for Animal Experiments at the University of Toyama. 

### 2.2. Perfusion and Tissue Processing

Under deep anesthesia with sodium pentobarbital (50 mg/kg body weight, i.p.), the mice were transcardially perfused with heparinized saline (0.9% w/v NaCl), followed by 4% paraformaldehyde dissolved in 0.1 M phosphate buffer (PB), pH 7.4. After perfusion, the brains were removed from the skull, cut coronally into small blocks and postfixed in 4% paraformaldehyde for overnight. The fixed brain blocks were immersed into 30% sucrose in 0.1 M PB until they sank down on the bottom. The brain blocks were freezed in dry ice and coronally cut into sections at the thickness of 40 *µ*m. The sections were collected in 0.01 M PBS and then transferred into a cryoprotectant solution (25% ethylene glycol, 25% glycerin, and 50% 0.1 M PBS) and stored at −20°C until immunohistochemical staining.

### 2.3. Immunohistochemistry of the Sections

Three serial sections were collected every 120 *µ*m, two for PV and Otx2-immunocytochemistry and one for Nissl staining with cresyl violet. The sections were washed thrice in 0.01 M phosphate buffer saline (PBS) for 15 min, blocked with 3% normal horse serum 0.25% Triton X-100 in PBS for 30 min at room temperature, and incubated overnight at 4°C with mouse monoclonal anti-PV (1 : 10,000 dilution, Sigma, St. Louis, MO, USA) or goat polyclonal anti-Otx2 (1 : 200 dilution, R&D) antibodies in 1% blocking solution 0.25% Triton X-100 in PBS. These sections were washed thrice with 0.01 M PBS for 5 min each time, incubated with biotinylated horse anti-mouse IgG or anti-goat IgG (1 : 250 dilution, Vector, Burlingame, USA) for 1 hr at room temperature, and then, after washing, incubated in ABC reagent (Vector) for 50 min. Finally, the PV- and Otx2-immunoreactive elements were visualized by reacting with 20 mg 3,3′-diaminobenzidine and 20 *µ*L 30% H_2_O_2_ in 100 mL 0.05 M PBS (pH 7.6) for 5–8 min. The sections were then rinsed several times in PBS, dehydrated in graded concentrations of ethanol, cleared in xylene, and cover-slipped with Entellan (Merck, Darmstadt, Germany). To minimize variability in staining, the sections from the all treatment groups were run in the same immunocytochemistry session. Negative control sections were treated identically except for omission of the primary antibody. No reaction product was observed in any of the control sections.

The corresponding adjacent sections were stained with cresyl violet to identify colliculus layers. The colliculus layers were identified by staining and cellular densities and morphologies of SC neurons [1]. We also identified collicular layers using parvalbumin-labeled sections where the collicular layers of these proteins have been well described previously [12, 52, 53].

### 2.4. Identification of the SC and Image Capture

In each section, the SC was identified and subdivided into collicular layers. The nomenclature of the mouse atlas of Patrick and Warren (2000) was adopted. Labeling was examined using standard bright-field microscope (BX 61; Olympus, Tokyo, Japan) with different magnifications (4~100x). A series of images of the SC were captured by a digital camera (DP 70, Olympus, Tokyo).

### 2.5. Data Analysis

 In the present study, 6 coronal sections (−3.30, −3.62, −3.94, −4.26, −4.58, and −4.90 mm from the bregma) in each mouse were analyzed for quantification of Otx2- and PV-IR cells. These digital images were analyzed using the ImageJ software (NIH ImageJ; http://rsbweb.nih.gov/ij/). The mean number of cells per section in each layer was estimated based upon two approache the measurement of pixel intensity and counting of immunopositive cells. For the pixel intensity of immunopositive cells, all images used for cell quantification were saved as uncompressed 8-bit grey-scale tiff files and then were thresholded using the autothreshold tool in ImageJ, with visual comparison being made to the original grey-scale images to ensure that the tool effectively resolved all labeled cell somas or nuclei. Therefore, particle analysis was carried out using the analyze particle tool. The mask tool was used to confirm that all particles (labeled cell somas or nuclei) were detected and measured.

Estimates of cell number in two-dimensional sections are subject to error and bias, which can be partially overcome by use of the Abercrombie correction [54, 55]. To enable such a correction in the current study, sections were cut at thickness of 40 *μ*m, and nuclear diameter of Otx2-IR cells and soma diameters of PV-IR cells (the average of long and short axes) were computed by measuring the long and short axes of cells counted. The correction factor was then applied to all cell measurements. The assessments were carried out by a single investigator (JJ Z), blind to group status. In order to furthermore diminish variability, rating of images was performed on 10 different occasions by the same blinded observer. Great care was taken to match sections through the same region of the brain and at the same level using the anatomical landmarks. Layers of the SC were identified using the counterstained sections of both the control and KO mice and then outlined using commercial software (Corel Draw 12). The collicular layers traced as above were analyzed on the corresponding collicular areas of immunocytologically processed control and KO mice. 

We analyzed the mean number of cells in each layer (6 sections from each of the 6 control mice and 4 KO mice) the diameter of each cell counted (6 sections from each of the 4 control mice and 4 KO mice), and categorized cells as vertical and horizontal fusiform, pyriform, marginal (round), and multipolar cells according to the previous studies [56, 57]. All statistical comparisons (*t*-test and repeated measure ANOVA) were performed using SPSS 17.0 software. A value of *P* < 0.05 was considered to be significant.

## 3. Results

### 3.1. Distribution of Otx2- and Parvalbumin (PV-) Immunoreactive (IR) Cells in the SC

**Figure 1:**
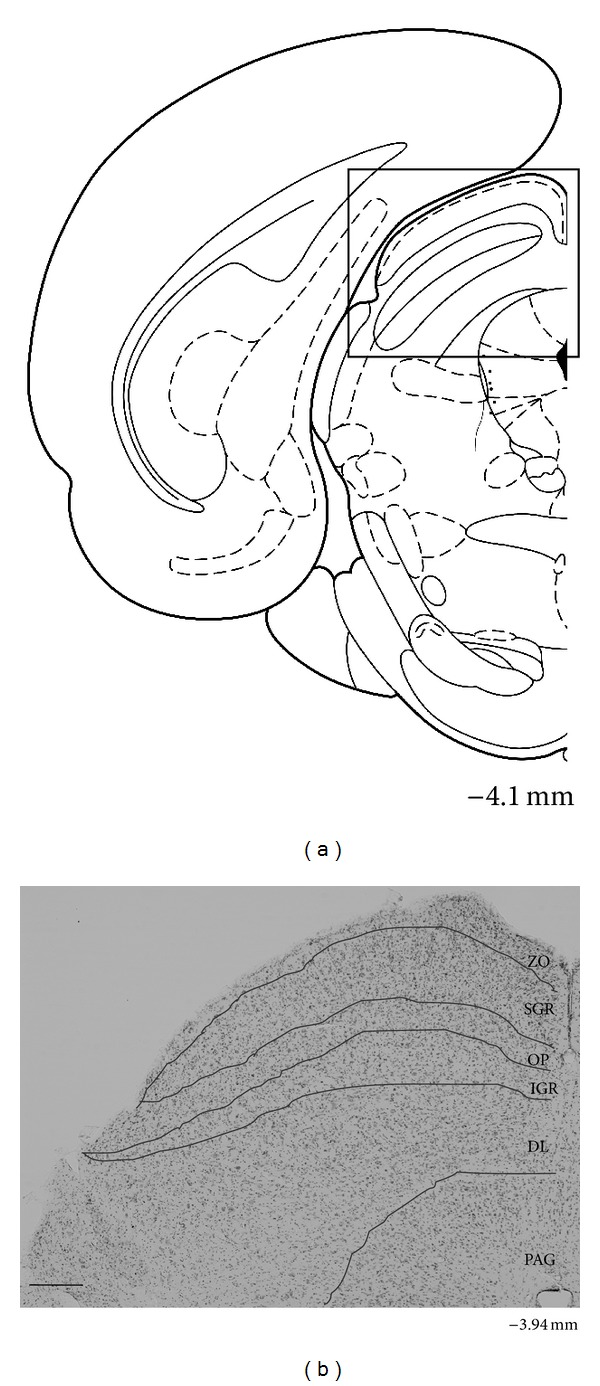
Coronal section in the brain atlas (AP, −4.1 mm from the bregma) (a) and representative section stained with cresyl violet (AP, −3.94 mm from the bregma) (b) showing the location and laminar pattern of the mouse SC. The square in A indicates the SC region. ZO zonale; SGR superficial gray layer; OP optic; IGR intermediate gray layer; DL, deep layer; and PAG periaqueductal gray matter. Scale bar = 200 *μ*m.

Figure 1 shows the location of the SC in the coronal (a) sections of the mouse brain atlas and a section stained by cresyl violet (b) indicating different subdivisions of the SC. Neuroanatomically, the SC has two divisions: a superficial subdivision comprising the zonal (ZO), superficial gray (SGR), and optic (OP) layers and a deep subdivision comprising the intermediate gray, intermediate white, deep gray, and deep white layers. The two layers of white matter in the deep subdivision are often rather indistinct and do not appear to demarcate cellular distributions. Therefore, we simply divided the deep subdivision into two regions: the intermediate gray (IGR) layer and the deep layers (DLs) including the deep white, deep gray, and intermediate white layers.

**Figure 2:**
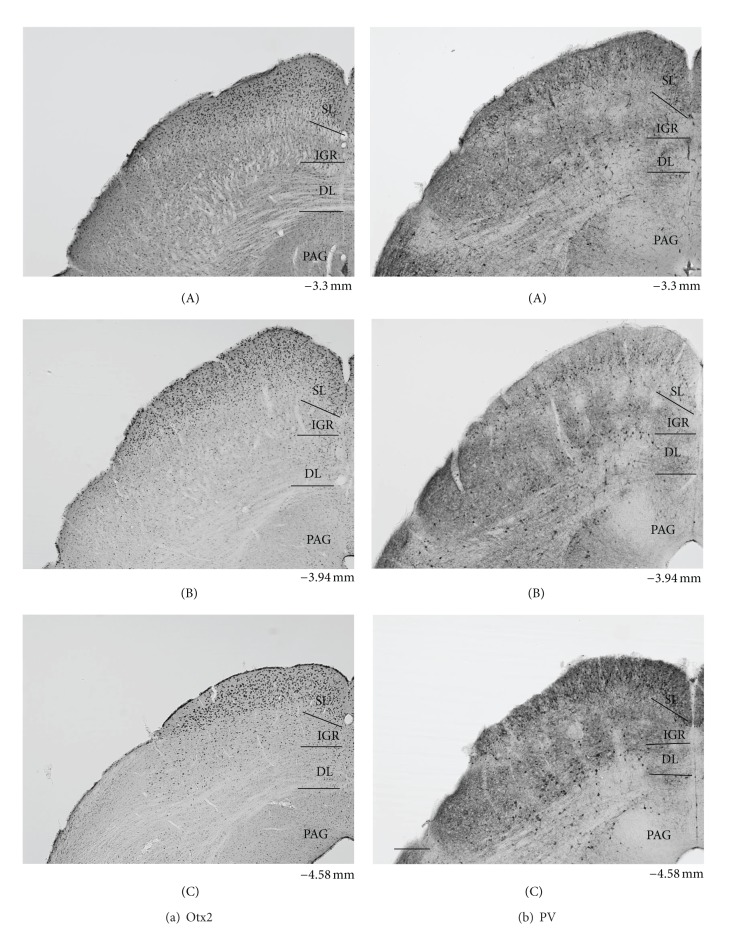
Low power photomicrographs of the laminar distribution of Otx2-IR (a) and parvalbumin (PV-) IR (b) elements in the control mouse SC. Two matching series of left coronal sections in 3 AP levels (A–C) are presented. Note prominent Otx2-IR cells in the superficial layers (SLs) and scattered Otx2-IR cells in the intermediate gray layer (IGR) and deep layers (DLs). Also note strong PV-labeling in the SL, patchy PVlabeling in the IGR, and scattered and/or clustered PV-IR cells in the IGR and DL. Number below each section indicate distance from the bregma. PAG periaqueductal gray matter. Scale bar = 200 *μ*m.

**Figure 3:**
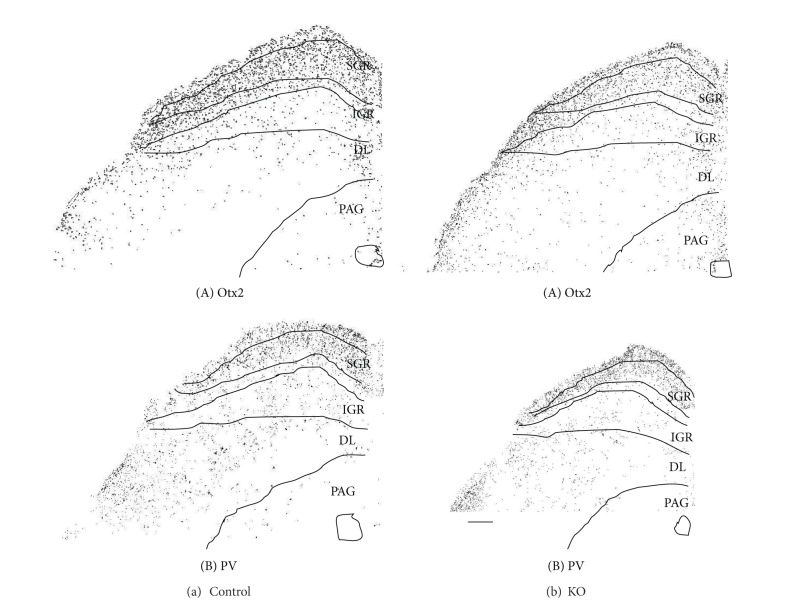
Distribution of Otx2-IR (A) and parvalbumin (PV-) IR (B) cells in the control (a) and KO (b) mice. Image data were masked by threshold intensity using ImageJ. SGR superficial gray; IGR intermediate gray layer; DL deep layer; PAG periaqueductal gray matter. Scale bar = 200 *μ*m.

**Figure 4:**
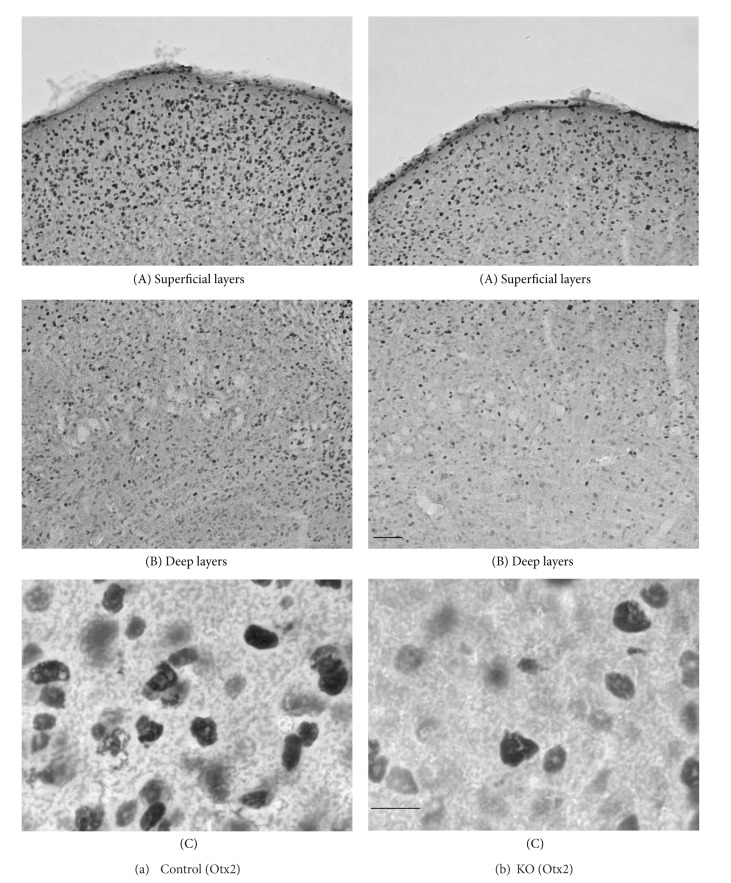
High-magnification photomicrographs of the Otx2-labeled sections in the control (a) and KO (b) mice. (a, b) Photomicrographs in the superficial (A) and deep main (B) layers of the SC. Note that the darklylabeled small-to-medium cells are densely distributed in the superficial layers in the SC of the control mouse. Similar patterns of distribution of Otx2-IR cells are also observed in the KO mice, but fewer Otx2-IR cells are observed in the KO mice. Scale bar = 60 *μ*m. (C) Higher-magnification photomicrographs of the Otx2-IR cells in the optic (OP) layer. Note oval- or round-shaped Otx2-IR cells. IRelements are localized in the nucleus of the cell body. Orientation of the images is dorsal to the top and lateral to the left. Scale bar = 10 *μ*m.

Figure 2 illustrates low power photomicrographs of the control mouse SC sections stained with Otx2 antibody (A) and PV antibody (B) indicating the laminar distributions of Otx2 and PV-IR cells.  Figure 3(a) shows black and white photomicrographs of the rostral SC sections stained with Otx2 (a) and PV (b) antibodies in the control mice as a function of the threshold intensity. The image data were thresholded to highlight the labeled cell somas or nuclei.  Figure 4(a) indicates high-magnification photomicrographs of the Otx2-labeled sections in the superficial (A) and deep main (B) layers of the SC in the control mice. The patterns of Otx2 and PV labeling in the present study were different from each other. Otx2-IR cells were found in all layers of the SC, but more densely in the superficial layers. PV-IR cells were also observed in all layers of the SC, but different from that of Otx2-IR cells (see below in detail).

The dense distribution of Otx2-IR cells was found in the superficial layers (ZO, SGR, and OP) (Figures 2(a), 3(a)(A), and 4(a)(A)). Otx2-IR cells were also observed in the deep layers (IGR, and DL) (Figures.  2(a), 3(a)(A), and 4(a)(B)), which mainly included lightly labeled, small to medium-sized cells (Figure 4(a)(B)). In contrast, very few labeled cells were observed in the areas surrounding the SC. The similar distribution patterns of Otx2-IR cells were observed through the rostral-caudal extent of the SC (Figure 2(a)).

**Figure 5:**
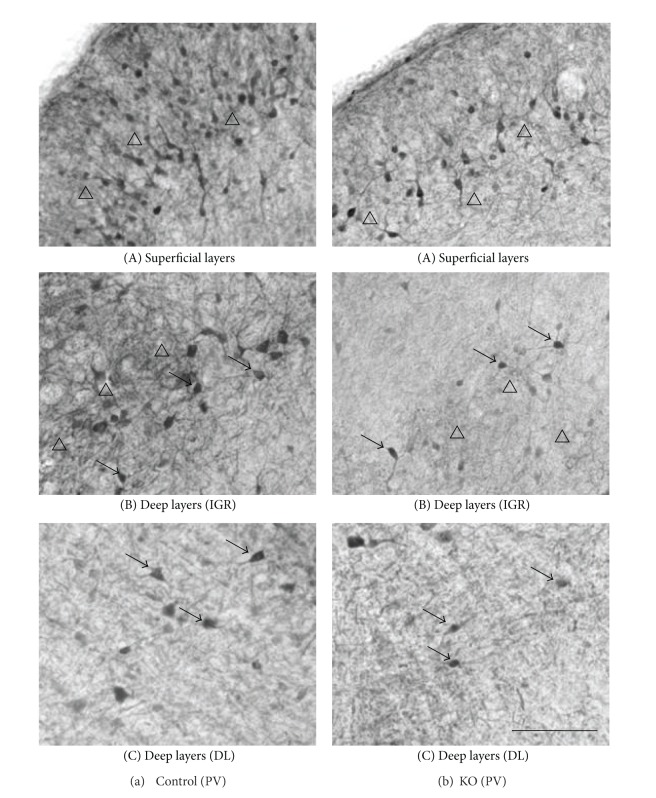
High-magnification photomicrographs of the PV-labeled sections in the superficial layers (A), IGR (B) and DL (C) of the control (A) and KO (B) mouse SC. Note a dense band in the deep SGR and the upper OP (open triangles) (A), clusters of PV-IR cells (arrows) and the intense PV-IR neuropils (open triangles) in the IGR (B), and scattered PV-IR cells (arrows) in the DL (C) in the control mouse (a). Similar patterns of distribution of PV-IR cells are also observed in the KO mice (b), but fewer PV-IR cells and less intense PV-IR neuropils are observed. Orientation of the images dorsal to the top and lateral to the left. SGR superficial gray; OP optic; IGR intermediate gray layer; is DL and the deep layers. Scale bar = 60 *μ*m.

Figure 5(a) indicates high-magnification photomicrographs of the PV-labeled sections in the superficial (A) and deep main layers (B, C) of the control mouse SC. PV-IR cells were concentrated within the superficial layers of the SC, with a heavier frequency within SGR than in the OP (Figure 5(a)(A). Also see Figures 2(b) and 3(a)(B)). Labeled IR elements in the dense tier included not only lightly stained small-to-medium sized somas but also proximal dendrites and small immunoreactive puncta that could be axons, axon terminals, or small dendrites (open triangles) (Figure 5(a)(A)). PV-IR cells were also scattered throughout the deep layers below the dense tier. In the lateral part of the IGR, clusters of PV-IR cells (arrows) were observed in the immunoreactive fiber patches (open triangles) (Figure 5(a)(B)). Scattered medium-to-large PV-IR cells (arrows) were found in the DL (Figure 5(a)(C)), including a few that are immediately adjacent to the periaqueductal gray and, occasionally, in the ZO. The neuropil labeling was patchy in the IGR and moderate in the DL (Figure 2(b)).

### 3.2. Morphology of the Otx2- and PV-IR Cells in the Control Mice

Figure 4(a)(C) represents various types of Otx2-IR cells in the control mouse SC. The principal cell types in the mouse SC labeled with Otx2 antibody were dark or light round to oval cells. These labeled IR elements were located in the nucleus of the cell body, consistent with a previous study [30, 58]. However, ratios of the strongly and weakly stained cells varied between the superficial and deep layers of the SC. In the superficial layers of the SC, a large majority of Otx2-IR cells were composed of the strongly stained cells. In the deep layers of the SC, most of the labeled cells were weaker and smaller compared with those observed in the superficial layers of the SC. Furthermore, there were some differences in diameter (the average of long and short axes) between the two divisions. The average diameter of Otx2-IR cells in the superficial layers of the SC ranged from 3.26 to 9.00 *μ*m with a mean of 6.01 *μ*m. The average diameter of Otx2-IR cells in the deep layers of the SC ranged from 3.17 to 8.50 *μ*m with a mean of 5.75 *μ*m. Statistical comparison indicated that the mean sizes of Otx2-IR cells were significantly larger in the superficial layers than those in the deep layers (unpaired *t*-test, *P* < 0.001).

**Figure 6:**
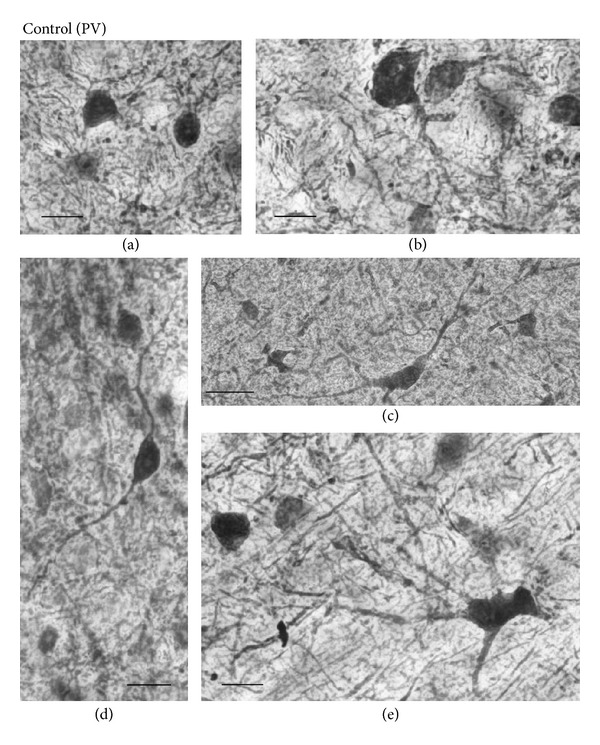
Representative examples of high-magnification photomicrographs of PV-IR cells in the control mouse SC. (a) Labeled soma of marginal PV-IR cells in the deep superficial gray (SGR). (b) Pyriform PV-IR cell in the PV-IR fiber patch in the lateral intermediate gray layer (IGR). (c) Horizontal fusiform cell in the lateral region of the deep layers (DL). (d) Vertical fusiform cell in the ventral SGR. (e) Large multipolar PV-IR cell in the DL. Orientation of the images is dorsal to the top and lateral to the left. Scale bars = 10 *μ*m.

Figure 6 represents various types of PV-IR cells in the control mouse SC. We categorized the PV-IR cells as vertical and horizontal fusiform, pyriform, marginal (round), and multipolar cells according to the previous studies [56, 57]. The types of PV-IR cells identified in the mouse SC were more variable than the classes of Otx2-IR cells. These labeled cells consisted of at least four distinct morphologies. Marginal cells had small, roundish, or stellate soma with few or no labeled dendrites (a). These cells were the most prominent type in the superficial layers of the mouse SC. Pyriform (pyramidal) cells had pear-shaped soma with one tufted dendritic tree extending above the cell body (b). This cell type was located mostly in the ventral SGR, IGR, and DL. 

Horizontal fusiform cells had horizontally elongated triangular or fusiform cell, with stout dendrites coursing in the same plane as the cell body (c). These cells were found mostly in the dorsal OP and in the lateral region of the DL. Vertical fusiform cells had vertical fusiform soma, usually with one dendrite emanating vertically from each pole (d). These cells were located mostly in the ventral SGR. 

Fourth type of the PV-IR cells was multipolar cells with large, lightly stained multipolar soma and several dendrites that radiated from the soma (e). These cells were observed in the IGR and DL. 

Furthermore, the three layers had their corresponding diameters. The average diameter of PV-IR cells in the superficial layers of the SC ranged from 6.08 to 21.62 *μ*m with a mean of 9.13 *μ*m. In the IGR, the diameter of labeled cells ranged from 6.08 to 20.27 *μ*m with a mean of 10.76 *μ*m. In the DL of the SC, the diameter of PV-IR cells ranged from 7.03 to 21.62 *μ*m with a mean of 12.91 *μ*m. Statistical analysis indicated that there was a significant difference in cell size among the 3 laminae (*P* < 0.001, Bonferroni tests after repeated measures one-way ANOVA).

### 3.3. Otx2 and PV Expression in the PDGFR-*β* KO Mice

Comparison of the control and KO mice revealed similar distribution patterns of Otx2- and PV-IR cells in the SC (Figure 3), in which the image data were thresholded to highlight the labeled cell somas or nuclei. Thus, two distinctive tiers of Otx2-IR labeling in the KO mouse SC (Figure 4(b)) were virtually identical to those in the control mice (Figure 4(a)). The band of densely labeled, small-to-medium-sized PV-IR cells in the superficial layers (SGR and OP) was also present in the KO mice (Figure 5(b)(A)). Clusters of PV-IR cells surrounded by the patchy fiber in the IGR were also visible in the KO mice (Figure 5(b)(B)). We also observed scattered medium-to-large PV-IR cells in the DL of the KO mouse SC (Figure 5(b)(C)). Tables 1 and 2 reveal mean number of Otx2- and PV-IR cells in each layer of the mouse SC. Cell counts in each layer indicated that frequency distributions of the labeled cells were similar in the control and KO mice (Tables 1 and 2).

**Figure 7:**
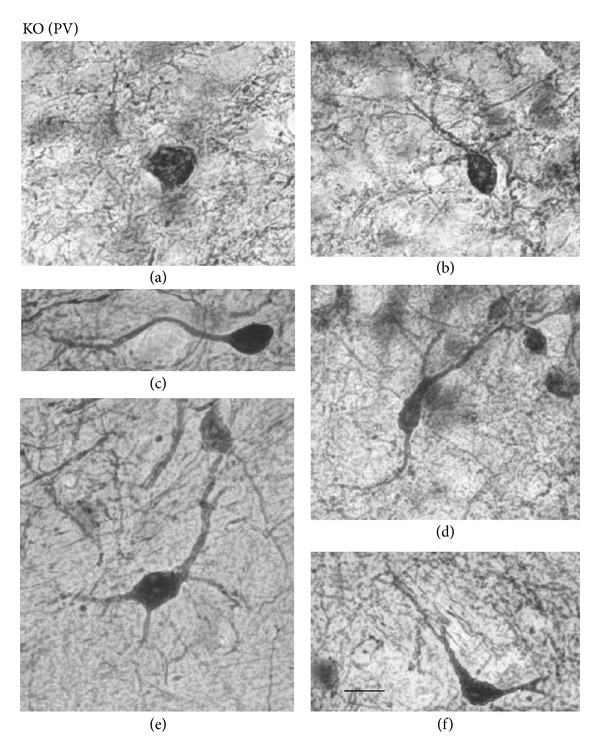
Representative examples of high-magnification photomicrographs of PV-IR cells in the KO mouse SC. (a) Marginal PV-IR cell in the deep superficial gray (SGR). (b), (c) Pyriform cells in the SGR and optic (OP), respectively. (d) Vertical fusiform cell in the SGR. (e): Multipolar cell in the deep layers (DLs). (f) Horizontal fusiform cell in the OP. Orientation of the images is dorsal to the top and lateral to the left. In all cases, scale bar = 10 *μ*m.

 Furthermore, Figures 4(b)(C) and 7 show high-magnification photomicrographs of Otx2- and PV-IR cells in the KO mice, respectively. All the various morphological Otx2- and PV-IR cell types in the control mice were also identified in the KO mouse SC. Thus, Otx2 antibody labeling in the KO mouse SC revealed dark or light round to oval cells (Figure 4(b)(C)). All the PV-IR cell types found in the SGR and OP in the control mice were also present in the KO mice (Figure 7). Marginal cells with star-shaped cell bodies and few or no labeled dendrites were commonly observed (a), and several pyriform cells were also found in the KO mouse SGR and OP (b, c). Vertical fusiform cells were also frequently seen in the SGR regions of the KO mice (d). Horizontal fusiform cells were observed in the KO mouse OP (f), each having a horizontally elongated cell body and two stout dendrites coursing from the cell body. The PV-IR cells in the KO mouse IGR were large and clustered (Figure 5(b)(B)), just as in the control mouse IGR. Finally, PV-IR cells in the KO mouse DL consisted of both multipolar (e) and horizontal fusiform (f) cells.

### 3.4. Effects of the PDGFR-*β* Gene on Otx2 and PV Expression

**Figure 8:**
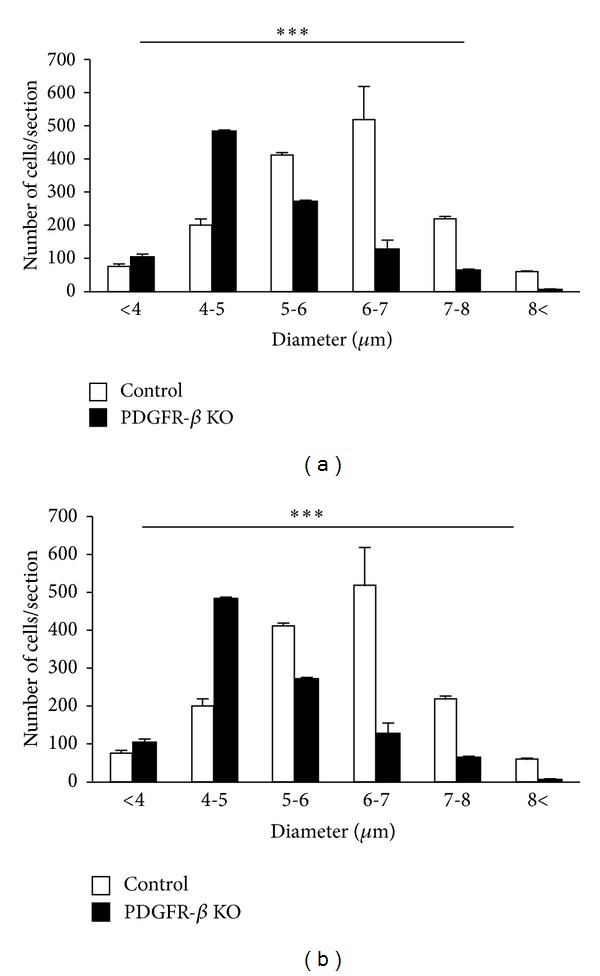
Frequency distributions of cell diameters of Otx2-IR (a) and PV-IR (b) cells in the SC of the control (open columns) and KO mice (black columns). Note that the average diameters of Otx2-IR and PV-IR cells in the SC were significantly larger in the control than those in KO mice. Error bars indicate standard deviation. *** Significant difference between the control and KO mice (*P* < 0.001, unpaired *t*-test).

Although the basic cell types and laminar distribution patterns of Otx2 and PV antibody labeling in the KO mice were similar to those in the control mice (see above), the following quantitative analyses indicated significant differences between the control and KO mice.  Figure 8(a) indicates distributions of average diameter of the Otx2-IR cells in the control and KO mouse SC. The average diameter of Otx2-IR cells in the whole control SC ranged from 3.17 to 9.00 *μ*m with a mean of 5.88 *μ*m.  Figure 8(b) revealed distribution of the diameter of PV-IR cells in the whole control SC ranging from 6.08 to 20.27 *μ*m with a mean of 10.85 *μ*m. The mean average diameters of Otx2- and PV-IR cells in the KO mouse SC were smaller than those in the control mouse (unpaired *t*-test, *P* < 0.001).

**Figure 9:**
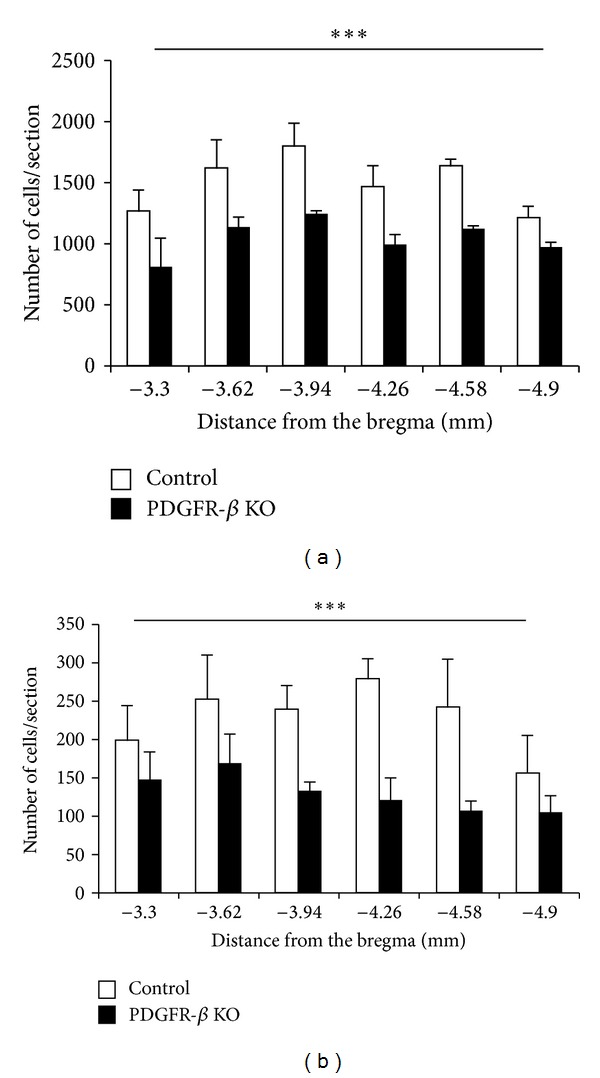
Comparison of mean number of Otx2-IR (a) and PV-IR (b) cells in the SC between the control (open columns) and KO mice (black columns). Each value represents the mean ± SEM. Numbers below the abscissa indicate the rostrocaudal distance from the bregma. Error bars indicate standard deviation. *** Significant difference between the control and KO mice (*P* < 0.001, repeated measures two-way ANOVA).

Figure 9(a) shows the number of Otx2-IR cells observed in the 6 coronal levels of the SC in the control and KO mice. Statistical analysis by repeated measures two-way ANOVA indicated that the mean number of the Otx2-IR cells in the whole SC was significantly reduced in the KO mice compared with the control mice *F*(1, 40) = 81.098, *P* < 0.001 (Figure 9(a)). This trend was also evident in the data processed by threshold mask in Figures 3(a)(A) and 3(b)(A). Mean numbers of the Otx2-IR cells per section in each lamina were also significantly decreased in the KO than those in control mice (unpaired *t*-test) (Table 1).

Figure 9(b) shows the number of PV-IR cells observed in the 6 coronal levels of the SC of the control and KO mice. Statistical analysis by repeated measures two-way ANOVA indicated that the mean number of the PV-IR cells in the whole SC was significantly reduced in the KO mice compared with the control mice *F*(1, 40) = 83.558, *P* < 0.001 (Figure 9(b)). This trend was also evident in the data processed by threshold mask in Figures 3(a)(B) and 3(b)(B). Statistical comparisons indicated that the average numbers of PV-IR cells were significantly decreased in each SC lamina of the KO mice except the ZO (unpaired *t*-test) (Table 2). Furthermore, analyses by pixel intensity also revealed similar changes (data not shown).

## 4. Discussion

The present study provides the first morphological and quantitative descriptions of Otx2-IR cells in the adult mouse SC. Immunohistochemical observations of the mouse SC in the present study demonstrated that Otx2-IR cells form two main tiers (superficial and deep layers with dense and light immunoreactivity, resp.), whereas PV-IR elements are concentrated in a dense tier in the superficial layer, which represent a pattern of sublimation in the mouse SC. In addition, this study provides the first evidence for the role of PDGFR-*β* in Otx2 and PV expression in the SC.

### 4.1. Otx2 Expression in the SC

The dense distribution of Otx2-IR cells is observed in the superficial layers of the mouse SC, and less frequent distribution was also found in the deep layers. Presence of Otx2 expression in the postnatal SC is consistent with previous studies in rats [30, 59]. In the present experiment, intensity of Otx2-IR labeling was different among the Otx2-IR cells. Transcription factors including Otx2 propagate along the visual pathway by trans-synaptic (cell-to-cell) transfer [30, 60], and the SC receives direct afferents from the retina where the Otx2 protein is strongly expressed [26, 30, 61]. These findings suggest that Otx2-immunoactivity in the Otx2-IR cells in the SC might be derived from the retinal ganglion cells. Therefore, strength of Otx2-immunoreactivity in individual SC neurons might be dependent on synaptic strength between the given SC neurons and retinal ganglion cells. Furthermore, a diversity of Otx2 proteins and thier mRNA isoforms has been reported in the mouse brain [62]. This finding also supports a diversity of Otx2-immunoreactivity in the SC. Further studies are required to clarify these phenomena.

The Otx2 expression in the brain is involved in visual information processing at both prenatal and postnatal stages [25–29, 63], and its role in differentiation of the retina [64, 65] has been well established. However, the exact function of Otx2 in the SC remains unclear. The superficial layers of the SC receive visual information from the retina and visual cortex and are involved in visual information processing [1, 4–6], while the deep SC layers play a role in integrating sensory information into motor signals that help to orient the head and body toward various stimuli and in saccadic eye movements [1, 4–6]. On the other hand, Otx2 is involved in the positioning of the isthmic organizer and control of neuronal subtype differentiation in the mid-brain during embryogenesis [60, 66, 67]. Furthermore, Otx2 is implicated in neural plasticity of PV-IR neurons in the postnatal visual cortex [30] and in axonal navigation and shaping neuronal arbors [60, 68]. These results suggest that Otx2 in the SC might be involved in formation and/or maintenance of precise topographical neuronal circuits such as retinotopic organization in the superficial layers [69, 70] and in formation and/or maintenance of neural circuits for spatially selective orientation of the body in the deep layers [71, 72].

### 4.2. PV Expression in the SC

PV-IR elements were rich in all collicular layers, and superficial gray (SGR) was homogeneously stained. The staining is attributed to rich varicose neuropils as well as a population of vertically oriented cells with a similar shape, which were more numerous in the dorsal optic (OP) than in the ventral SGR. In contrast to the staining pattern in the superficial layers, the pattern of immunoreactivity in the IGR was clustered. This pattern appears as a row of patches with various holes of different sizes, in which numerous marginal and multipolar cells are mostly found. The similar PV-immunolabeling patterns described in the present study are also reported in the rat SC [14]. In the superficial layers of the SC, a diffuse superposition by both PV- and Otx2-IR substances was observed, suggesting that some Otx2-IR cells coexpress PV protein. Further studies using double-labeled staining are required to confirm colocalization of Otx2- and PV-IR cells in the adult mouse SC.

In the SC, about 50% of PV-IR cells were GABAergic, and GABAergic neurons in the SC are small to medium in size (less than 15 *μ*m mean diameter) [73]. These findings are consistent with present studies in which PV-IR cells with the same size were observed. These GABAergic are involved in both intralaminar and interlaminar inhibition [74]. Consistently electrical stimulation within the superficial layers in the SC evoked inhibitory postsynaptic current [75], and projection from the superficial layers to deep layers in the SC is under tonic inhibition by GABAergic activity [74]. These GABAergic circuits are suggested to play a role in shaping receptive field inhibitory surrounds as well as visual response habituation [76] and sensorimotor processing for orientation [74]. Furthermore, PV-IR interneurons with fast spiking are involved in gamma oscillation [77, 78], which is induced in the SC by visual stimulation [79, 80]. These findings suggest that PV-IR cells are involved in gamma oscillation in the mouse SC. 

On the other hand, the remaining half of the PV-IR cells in the SC might be excitatory and colocalize with glutamate [73, 81]. These PV-IR excitatory neurons could be excitatory projection neurons with large cell size [81] or local interneurons in the excitatory networks to generate high-frequency burst activity in premotor neurons in the intermediate layers [73, 82] since these premotor neurons in the intermediate layers have no intrinsic mechanisms to support high-frequency burstic activity [6].

### 4.3. Role of PDGFR-*β* in PV and Otx2 Expression the SC

PDGF-B chain together with expression of PDGFR-*β* can widely exert a neurotrophic or regulatory activity on neurons in an autocrine manner or by neuron to neuron interactions in the brain [83]. In the present study, we observed significant reductions in Otx2- and PV-IR cells in the SC of the KO mice. Furthermore, cell sizes of Otx2- and PV-IR cells were significantly reduced in the SC. Since Otx2-immunoreactive elements are usually localized in the nucleus of the cell body, decrease in Otx2-IR cell size might reflect decrease in expression of Otx2 protein. These findings suggest that PDGFR-*β* exerts substantial effects on expression of Otx2 and PV in the SC.

Since PDGF-B/PDGFR-*β* signal axis exerts neurotrophic effects on GABAergic neurons [42, 45], a decrease in the number of PV-IR cells might be attributed to the loss of neurotrophic effects of PDGFR-*β* in the PDGFR-*β* KO mice. Furthermore, Otx2 and PDGF-B/PDGFR-*β* exert similar effects on neurons; both Otx2 and PDGF-B exert neurotrophic effects on dopaminergic neurons [67, 68, 84], are involved in maturation of GABAergic neurons [30] or exert neurotrophic effects on GABAergic neurons [42, 45], and are also involved in neurogenesis and migration of some neurons [51, 67, 85]. These results suggest that effects of PDGFR-*β* might be partially mediated through Otx2. Consistent with this idea, Otx2 expression is controlled by neurotrophic factors such as basic fibroblast growth factor (bFGF) [86], and PDGFR-*β* signals are indispensable for the bFGF signals [51]. The present results along with these previous findings suggest that PDGF-B might directly affect development of PV-IR cells, and/or indirectly affect development of PV-IR cells through its effects on Otx2. Further studies are required to elucidate signal transduction pathways among PDGFR-*β*, Otx2 and PV. 

## 5. Conclusions

The present study demonstrates the distribution patterns of Otx2- and PV-IR cells in the mouse SC. Both the Otx2-IR cells and PV-IR elements revealed characteristic patterns. The labeled cells were heterogeneous in diameter. Although these characteristic patterns did not differ between the control and PDGFR-*β* KO mice, mean numbers of the Otx2- and PV-IR cells were significantly decreased in the PDGFR-*β* KO mice. Furthermore, Otx2- and PV-IR cell sizes were significantly smaller in the PDGFR-*β* KO mice. These findings suggest that PDGFR-*β* KO induces development and maturation deficits in Otx2- and PV-IR cells in the SC. These results further suggest that behavioral traits (socioemotional deficits) of PDGFR-*β* KO mice [48] might be ascribed partly to deficits in PV-positive GABAergic neurons in the SC of PDGFR-*β* KO mice. This idea is supported by previous studies; bilateral SC lesions reduced social behaviors [7, 8] and reduction of number of PV-containing GABAergic neuronsin the SC is associated with autism and schizophrenia with social deficits [17–19]. Activity in the subcortical visual pathway including the SC was altered in autistic patients [87]. Further studies are required to elucidate molecular mechanisms of the role of PDGFR-*β* in survival and induction of Otx2 and PV proteins.

## Figures and Tables

**Table 1:** Comparison of number of Otx2-IR cells in the SC between the control (C1–6) and PDGFR-*β* KO (K1–4) mice.

SC subfield	Control							PDGFR-β KO						
	No. of cells per section							No. of cells per section						
	C1	C2	C3	C4	C5	C6	Mean ± SD	K1	K2	K3	K4	Mean ± SD	%Change	*P* value
ZO	224.8	214.0	246.2	203.8	223.3	234.8	224.5 ± 15.0	153.8	157.5	160.8	156.8	157.2 ± 2.9	−30.0%	0.001
SGR	378.8	358.7	376.0	401.0	391.8	454.2	393.4 ± 33.1	240.5	271.2	231.0	239.3	245.5 ± 17.6	−37.6%	0.001
OP	325.5	385.7	399.0	330.3	350.0	354.7	357.5 ± 29.5	269.3	280.0	247.5	261.0	264.5 ± 13.7	−25.2%	0.001
IGR	316.2	316.2	302.0	279.2	328.5	299.8	307.0 ± 17.2	214.7	229.0	220.7	216.7	220.3 ± 6.3	−28.2%	0.001
DL	189.8	168.8	258.2	159.2	268.7	272.3	219.5 ± 52.5	176.3	138.3	147.0	159.2	155.2 ± 16.5	−29.3%	0.048

For each layer of the SC, the mean numbers of cells per section in each animal are indicated. In each animal, 6 sections were examined. %Change and *P* value indicate %change from the control mice and statistical significance from the control mice (unpaired *t*-test) in each layer. ZO: zonal layer; SGR: superficial gray layer; OP: optic layer; IGR: intermediate gray layer; and DL: deep layer.

**Table 2:** Comparison of number of PV-IR cells in the SC between the control (C1–6) and PDGFR-*β* KO (K1–4) mice.

SC subfield	Control							PDGFR-*β* KO						
	No. of cells per section							No. of cells per section						
	C1	C2	C3	C4	C5	C6	Mean ± SD	K1	K2	K3	K4	Mean ± SD	%Change	*P* value
ZO	4.0	9.8	5.3	9.2	7.0	8.0	7.2 ± 2.2	5.8	9.5	7.2	7.7	7.5 ± 1.5	4.2%	0.804
SGR	52.3	54.5	77.5	49.5	85.8	54.0	62.3 ± 15.3	41.2	31.5	29.8	31.5	33.5 ± 5.2	−46.2%	0.007
OP	76.0	66.2	78.8	71.7	87.3	65.5	74.3 ± 8.3	43.5	38.5	45.3	40.5	42.0 ± 3.0	−32.7%	0.001
IGR	33.0	23.5	28.3	23.0	24.8	24.3	26.2 ± 3.8	15.8	16.5	15.5	13.5	15.3 ± 1.3	−41.6%	0.001
DL	55.5	55.0	51.7	61.8	58.7	63.5	57.7 ± 4.5	25.2	29.2	34.7	33.0	30.5 ± 4.2	−47.1%	0.001

For each layer of the SC, the mean numbers of cells per section in each animal are indicated. In each animal, 6 sections were examined. %Change and *T* value indicate the %change from the control mice and statistical significance from the control mice (unpaired *t*-test) in each layer. For abbreviations, see Table 1.
